# Effect of Copper-Modification of g-C_3_N_4_ on the Visible-Light-Driven Photocatalytic Oxidation of Nitrophenols

**DOI:** 10.3390/molecules28237810

**Published:** 2023-11-27

**Authors:** Truong Nguyen Xuan, Dien Nguyen Thi, Quang Tran Thuong, Tue Nguyen Ngoc, Khanh Dang Quoc, Zsombor Molnár, Shoaib Mukhtar, Erzsébet Szabó-Bárdos, Ottó Horváth

**Affiliations:** 1 School of Chemistry and Life Sciences, Hanoi University of Science and Technology, No.1 Dai Co Viet Street, Hai Ba Trung District, Hanoi 100000, Vietnam; truong.nguyenxuan@hust.edu.vn (T.N.X.); quang.tranthuong@hust.edu.vn (Q.T.T.); tue.nguyenngoc@hust.edu.vn (T.N.N.); 2 Viettel Aerospace Institute, Viettel Group, Hoa Lac High-Tech Park, Thach That District, Hanoi 10000, Vietnam; diennt15@viettel.com.vn; 3 School of Materials Science and Engineering, Hanoi University of Science and Technology, No.1 Dai Co Viet Street, Hai Ba Trung District, Hanoi 100000, Vietnam; khanh.dangquoc@hust.edu.vn; 4 Environmental Mineralogy Research Group, Research Institute of Biomolecular and Chemical Engineering, University of Pannonia, P.O. Box 1158, H-8210 Veszprém, Hungary; molnar.zsombor@mk.uni-pannon.hu; 5 Research Group of Environmental and Inorganic Photochemistry, Center for Natural Sciences, Faculty of Engineering, University of Pannonia, P.O. Box 1158, H-8210 Veszprém, Hungaryszabone.bardos.erzsebet@mk.uni-pannon.hu (E.S.-B.)

**Keywords:** graphitic carbon nitride, photocatalysis, copper-modification, oxidation of nitrophenols, visible-light irradiation, pH effect, H_2_O_2_ effect

## Abstract

Graphitic carbon nitride (g-C_3_N_4_) has proved to be a promising heterogeneous photocatalyst in the visible range. It can be used, among others, for the oxidative conversion of environmentally harmful nitrophenols occurring in wastewater. However, its photocatalytic activity needs to be enhanced, which can be achieved by modification with various dopants. In our work, copper-modified g-C_3_N_4_ was prepared by ultrasonic impregnation of the pristine g-C_3_N_4_ synthesized from thiourea. The morphology, microstructure, and optical properties of the photocatalysts were characterized by XRD, FT-IR, DRS, SEM, XPS, and TEM. DRS analysis indicated a slight change in both the CB and the VB energies of Cu/g-C_3_N_4_ compared to those of g-C_3_N_4_. The efficiency of the photocatalysts prepared was tested by the degradation of nitrophenols. Copper modification caused a sevenfold increase in the rate of 4-nitrophenol degradation in the presence of H_2_O_2_ at pH = 3. This dramatic enhancement can be attributed to the synergistic effect of copper and H_2_O_2_ in this photocatalytic system. A minor Fenton reaction role was also detected. The reusability of the Cu/g-C_3_N_4_ catalyst was demonstrated through five cycles. Copper-modified g-C_3_N_4_ with H_2_O_2_ proved to be applicable for efficient visible-light-driven photocatalytic oxidative degradation of nitrophenols.

## 1. Introduction

From the advanced oxidation processes (AOPs), heterogeneous photocatalysis proved to be an efficient solution for the removal of several types of contaminants in wastewater [1]. One of the significant advantages of heterogeneous photocatalysts, mostly photoactive semiconductors, is that they can utilize solar radiation as a renewable energy (light) source. However, a considerable part of the efficient and stable semiconductor-type photocatalysts (such as TiO_2_, ZnS, SnO_2_, ZnO, etc.) cannot be excited in the visible range [2], which represents the determinant fraction of the solar light reaching the surface of Earth. Accordingly, colored semiconductors attract increasing interest in a better utilization of solar radiation. A significant part of such photocatalysts are organic, which are mostly molecular, but heterogeneous ones have also been developed and applied in more and more systems. Their metal-free structure is advantageous in respect of environmental considerations. In addition, the preparation of organic semiconductors (OSCs) is rather easy under relatively mild conditions. Graphitic carbon nitride (g-C_3_N_4_), which is a conjugated polymer, is considered to be a promising OSC [3]. It proved to be efficient in the utilization of light in the visible range due to its relatively narrow band gap (2.7–2.8 eV) [4,5]. Nevertheless, its visible-light response is rather limited (up to c.a. 450 nm), and the photogenerated charge carriers in this semiconductor undergo fast recombination, which also contributes to its drawbacks [6]. The combination of this OCS with various materials as dopants such as metals [7,8], non-metals [9,10], and other semiconductors [11,12] could offer good solutions to these challenges. Application of these modified g-C_3_N_4_ catalysts has been very diverse, for both oxidation and reduction of different substrates, e.g., for degradation of organic pollutants [13,14], CO_2_ reduction [15], and water splitting [16,17]. For the former purpose, copper-doped g-C_3_N_4_ was successfully applied in the removal of various antibiotics [18] such as levofloxacin [19], norfloxacin [20], and other pharmaceuticals [21], as well as pesticides [22] and various dyes such as methylene blue [23,24] and methyl orange [25].

Nitrophenols have been identified as hazardous pollutants that have been proposed for inclusion on the EPA National Priorities List [26]. These compounds are used as intermediates to produce dyes, pigments, pharmaceuticals, rubber chemicals, lumber preservatives, photographic chemicals, pesticides, and fungicides. Nitrophenols are released into the environment primarily from manufacturing and processing industries, as well as vehicle exhaust. Nitrophenols induce substantial changes in cell membranes and result in the development of various diseases through cytotoxicity [27].

Elimination of nitrophenols from various wastewaters has mostly been realized by adsorption using different kinds of adsorbents such as sand [28], calix [6] arene-tethered silica [29], or activated carbon developed from demineralized kraft lignin [30]. Besides this method, nitrophenols, especially 4-nitrophenol (4-NP), can be successfully degraded by photocatalysis based on the application of various g-C_3_N_4_ catalysts. Accordingly, the combination of g-C_3_N_4_ with MgO [31], ZnO [32], ZnBi_2_O_4_ [33], CuO with Cl^−^ [34], Fe_2_O_3_ [35], and an amine metal–organic framework [36] proved to be promising in this respect. Nevertheless, g-C_3_N_4_ was also utilized for this purpose without modification, although under UV irradiation [37].

The previous results related to the g-C_3_N_4_-based photocatalytic degradation of nitrophenols unambiguously indicate that numerous types of modifications can improve the efficiency of this process. In spite of the wide range of studies, however, the effect of the modification with copper and the application of such a photocatalyst in the presence of H_2_O_2_ has not been investigated for this purpose. Hence, the main goal of this work was the preparation and characterization of copper-modified g-C_3_N_4_ (designated as Cu/g-C_3_N_4_) and its application for the photocatalytic oxidative degradation of nitrophenols, especially 4-nitrophenol, compared to the efficiency achieved with the pristine g-C_3_N_4_. Optimization of the photocatalytic conditions (e.g., pH, component concentrations) as well as reusability experiments were also carried out. Our results unequivocally demonstrated that copper modification of the catalyst and its application with H_2_O_2_ dramatically improved the photocatalytic performance regarding the degradation of nitrophenols, and its efficiency hardly dropped through several reuse cycles.

## 2. Results and Discussion

### 2.1. XRD Analysis

**Figure 1:**
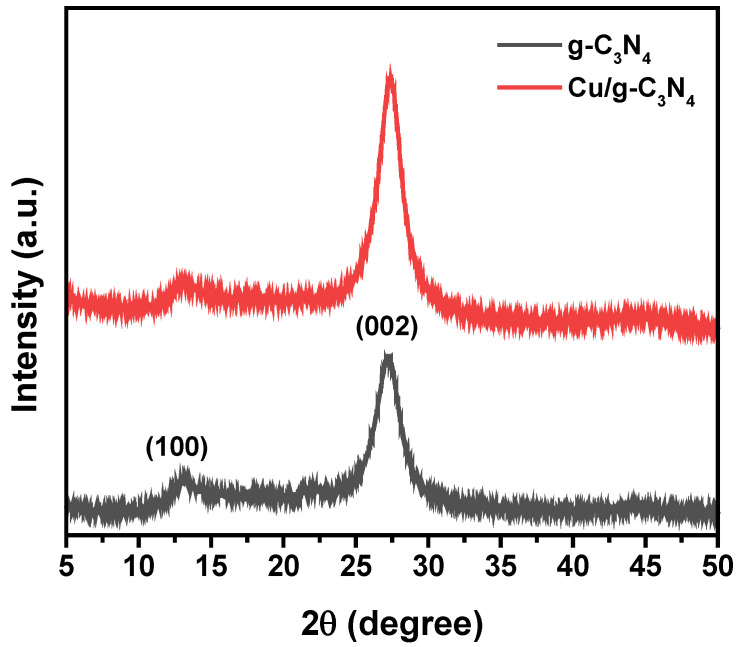
XRD pattern of g-C_3_N_4_ and 3% Cu/g-C_3_N_4_.

The XRD patterns of the Cu/g-C_3_N_4_ and g-C_3_N_4_ catalysts prepared in this work are displayed in Figure 1. The XRD patterns clearly demonstrate that Cu/g-C_3_N_4_ keeps the crystal characteristic of pristine porous g-C_3_N_4_. The weak peak that appeared at 13.08° and the intense one located at 27.56° are assigned to the (100) and (002) planes of g-C_3_N_4_ (JCPDS No. 87-1526), respectively [38]. The characteristic peaks at 27.56° are ascribed to the dense interlayer-stacking (002) peak of aromatic parts of g-C_3_N_4_ materials. The (002) diffraction of the carbon in Cu/C suggests that it possesses a quasi-graphitic structure. The reflection at 13.08° is marked as a (100) peak that originates from the in-plane ordering of tri-*s*-triazine attributed to units of g-C_3_N_4_. According to these results, the copper doping did not modify the basic structure of g-C_3_N_4_. The lack of an apparent characteristic peak of Cu on Cu/g-C_3_N_4_ suggests that copper particles have a small size and low loading, and they are well-dispersed on the g-C_3_N_4_ surface. This conclusion has been confirmed by the SEM and TEM analysis (see later).

### 2.2. SEM–EDS Analysis

**Figure 2:**
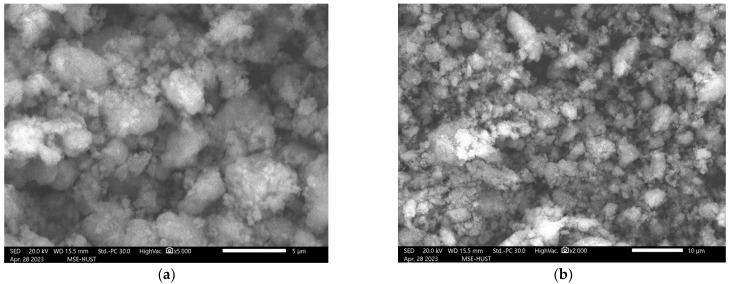
SEM images of catalyst materials Cu/g-C_3_N_4_. (**a**) Higher resolution (5000× magnification). (**b**) Lower resolution (2000× magnification).

The morphology of Cu/g-C_3_N_4_ consists of large (1–6 μm) agglomerates which are formed by the association of nanoparticles of less than 100 nm (Figure 2). No distinct copper nanoparticles could be detected on the surface of the Cu/g-C_3_N_4_ particles, indicating that the size of the copper species is very small, and these particulates (clusters) are well-dispersed on the catalyst surface. The SEM image of the unmodified catalyst displays nanoparticles of about the same size as in the case of the 3% Cu/g-C_3_N_4_ sample (Figure S1). In addition, between the catalyst particles, a considerable volume of voids can be observed, which ensures good accessibility to the active sites on the surface. This is confirmed by the adsorption efficiency of both types of catalysts, as indicated later in the first figure of Section 2.8.1.

The presence of C, N, and Cu in the 3% Cu/g-C_3_N_4_ sample can be confirmed using energy dispersive spectroscopy (EDS). The results of the EDS analysis (Figure S2 in Supplementary Materials) unambiguously indicated the presence of copper on the surface of the Cu/g-C_3_N_4_ catalyst. Even if it is a rather semi-quantitative method, the order of magnitude of the determined copper content (1.6 wt.%) is in accordance with the theoretical value.

### 2.3. FT-IR Analysis

**Figure 3:**
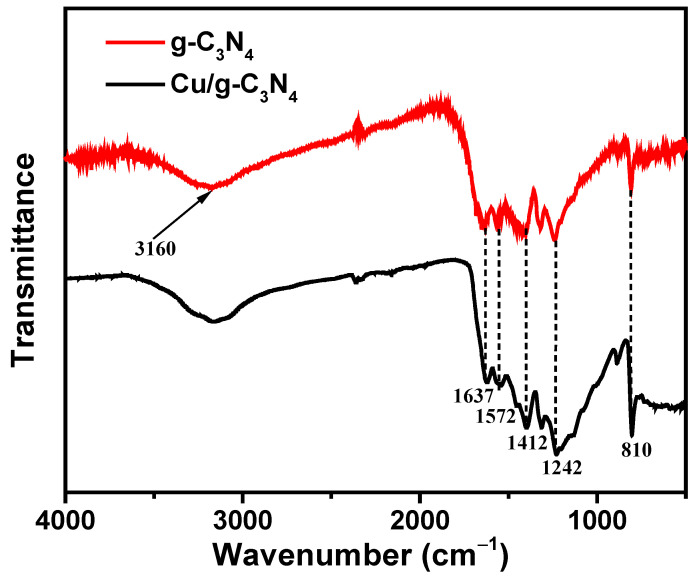
FT-IR spectra of 3% Cu/g-C_3_N_4_ (red line) and g-C_3_N_4_ (black line).

Figure 3 shows the FT-IR spectrum of the g-C_3_N_4_ and Cu/g-C_3_N_4_ catalysts. The characteristic absorption fringes at 3160 cm^−1^ on the IR spectra of g-C_3_N_4_ and Cu/g-C_3_N_4_, respectively, are ascribed to the valence oscillations of N–H [39]. Absorption fringes at about 1637 cm^−1^ and 1242 cm^−1^ can be related to the fluctuations of the C–N, C=N valences of the aromatic heterocyclic [40]. The intense bands at 1637, 1572, 1412, and 1242 cm^−1^ are ascribed to typical stretching vibration modes of triazine-derived repeating units. Finally, the strong absorption fringes at 810 cm^−1^ in both catalysts feature the oscillation of the s-triazine ring absorption band. In addition, there is no absorption pattern related to sulfur bonds (such as –SH, –SN, and –SC), indicating that elemental sulfur is completely liberated during heat treatment [41]. The spectra unambiguously show that all modes of vibration were preserved after copper incorporation, without any modification of the typical molecular structure of g-C_3_N_4_.

### 2.4. XPS Analysis

X-ray photoelectron spectroscopy was applied to check the surface ratio along with the chemical state of the elements in the modified photocatalyst, with a special emphasis on copper. The results of the XPS measurements confirmed its presence on the surface of 3% Cu/g-C_3_N_4_ with a content of 3.7% (for data see Table S1; for the C 1s and N 1s binding energy spectra, see Figures S3 and S4). This value is more than two times higher than that determined by SEM–EDS analysis. This deviation originates from the different penetrations of copper(II) ions during the impregnation and from the measuring depths of the two methods (3–10 nm for XPS; 3 μm for EDS). The C/N ratio of ca. 4.0/5.2 was consistent with the expected composition of carbon nitride. The analysis of the Cu 2p binding energy region revealed that satellites typical for Cu(II) were absent, hence this oxidation state could be excluded. The Cu 2p_3/2_ and Cu 2p_1/2_ peaks were present at 952.5 and 932.8 eV, respectively (Figure S5). The modified Auger parameter (Cu 2p_3/2_, L_3_M_45_M_45_) of 1847.8 clearly confirmed the Cu(I) instead of the Cu(0) oxidation state, as the corresponding parameter for the latter would be 1851.2 [42,43] (Figure S6). These results suggest that copper is present as a Cu(I)-C_3_N_4_ surface complex species in our decorated samples [44].

### 2.5. TEM Analysis

**Figure 4:**
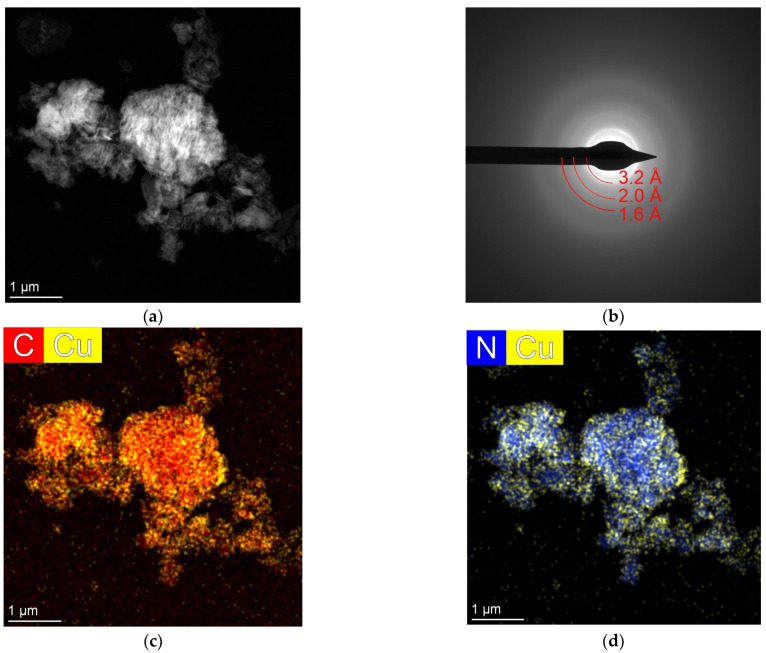
STEM micrographs of the 3% Cu/g-C_3_N_4_ sample. STEM HAADF image (**a**) of the assemblies of substrate grains with the corresponding selected area diffraction (SAED) pattern (**b**), where the measured *d*-values on the diffuse, ring-like intensity maxima (red) indicate the presence of graphite-related ordering in the substrate material. STEM EDS elemental maps (**c**,**d**) revealed small heterogeneities with respect to the Cu distribution.

In the Cu/g-C_3_N_4_ samples, we observed C- and N-containing grains with a typically large lateral size (few μm-s) but much smaller thickness; thus, we refer to them as C–N flakes (Figure 4a) The SAED patterns of the C–N flakes (Figure 4b) showed diffuse, ring-like intensity maxima, and their corresponding *d*-values (3.2 Å, 2.0 Å and 1.6 Å) indicate that the flakes were non-crystalline. Nevertheless, their *d*-values were close to the values of hexagonal graphite (3.34 Å, 2.02 Å and 1.67 Å), indicating some kind of graphite-related ordering in their structure. These larger flakes were probably formed by the agglomeration of several tens of nm particles. The STEM EDS maps and the corresponding EDS spectra of the graphitic C–N flakes showed a small Cu content (with about 0.52 atomic %, Figure S7), but we did not observe individual Cu nanoparticles (Figure 4c,d). The order of magnitude of the copper ratio is in accordance with the result obtained by the SEM–EDS analysis. However, the spatial distribution of Cu content on the surface of the C–N flakes showed small heterogeneities.

### 2.6. DR/UV-Vis Analysis Analysis

**Figure 5:**
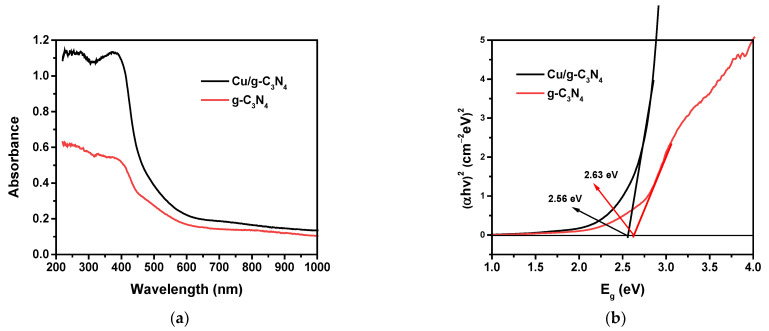
(**a**) DR/UV-Vis spectra; (**b**) energy bandgap determination by the Tauc plots for the catalysts: g-C_3_N_4_ and 3% Cu/g-C_3_N_4_.

Absorption spectroscopy is applied to determine the optical properties of the synthesized samples, providing important pieces of information related to light absorption by the heterogeneous photocatalysts. Figure 5 displays the DR/UV-Vis absorption spectra and the Tauc plot [45] of the prepared Cu/g-C_3_N_4_ and g-C_3_N_4_ samples. The considerable decrease in the absorption upon Cu-doping (Figure 5a) can be attributed to the darker color. At the same time, the reflection correspondingly decreased.

When doping Cu NPs onto the material, the band gap (Eg) decreased from 2.63 to 2.56 eV (Table 1), corresponding to the red shift of the absorption edge from 471 nm (g-C_3_N_4_) to 484 nm Cu/g-C_3_N_4_). Nevertheless, both catalysts showed absorption in the visible region, corresponding to the electron transition from the valence band (VB) to the conduction band (CB).

The values of E_VB_ and E_CB_ were estimated by the adoption of the Mulliken electronegative principle, using Equations (1) and (2):

E_CB_ = E_VB_ − E_g_ (1)

E_VB_ = χ − E_e_ + 0.5E_g_ (2)

where χ represents the Mulliken electronegative symbol of g-C_3_N_4_ (4.67 eV), and E_e_ is the energy of free electrons on the hydrogen scale (E_e_ ≈ 4.50 eV).

The same electronegativity (χ) was applied in this calculation for both g-C_3_N_4_ and Cu/g-C_3_N_4_ because Cu-doping just slightly modified it. According to this estimation, the E_CB_ value obtained for the modified semiconductor is a bit less negative than that of g-C_3_N_4_, while the E_VB_ is a bit less positive. Therefore, the band gap of the modified photocatalyst is narrower, and, thus, it can be excited at somewhat longer wavelengths in the visible range. However, the slight shifts in the valence and conduction bands, and, thus, the very modest narrowing of the band gap cannot cause a perceptible change in the photocatalytic efficiency as shown later (in Section 2.8.1).

### 2.7. Photocatalytic Activities

**Figure 6:**
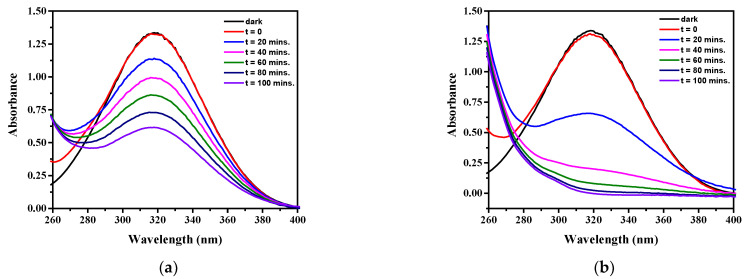
Spectral changes in 4-NP during the photodegradation by (**a**) g-C_3_N_4_ and (**b**) 3% Cu/g-C_3_N_4_ catalysts (20 ppm 4-NP, 10 mM H_2_O_2_, 1.0 g/L catalyst, pH 3).

The degradation of the 4-NP was spectrophotochemically followed; the characteristic absorbance at 320 nm decreased proportionally to the converted 4-NP concentration. Since no shift in this band was observed (see Figure 6), the intermediates formed in the degradation of the model pollutant do not absorb at 320 nm (and at longer wavelengths). Under the applied experimental conditions, during a 100 min irradiation period, in the presence of pristine g-C_3_N_4_, the conversion of 4-NP was about 53% (Figure 6a), while in the presence of the copper-modified catalyst (3% Cu/g-C_3_N_4_), practically total degradation was reached (Figure 6b). Of course, as shown by the temporal spectral changes, the difference between the degradation rates was much higher (about one order of magnitude, as indicated later by the corresponding rate constants). These spectral series for the demonstration were recorded under optimized experimental conditions, which were determined in the following investigations.

### 2.8. Optimization of the Experimental Conditions for 4-NP Photodegradation

#### 2.8.1. Effect of Copper Modification and the Presence of H_2_O_2_

**Figure 7:**
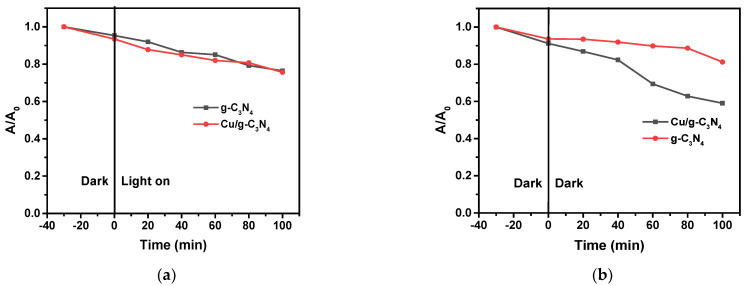

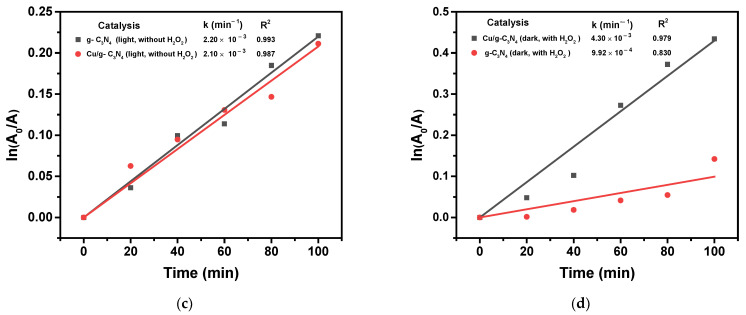
Degradation of 4-NP under different experimental conditions: (**a**) light; without H_2_O_2_; (**b**) dark; with H_2_O_2_ (10 mM) (1.0 g/L catalyst, pH 3); the corresponding logarithmic curves for the determination of rate constants, (**c**) from (**a**), (**d**) from (**b**). The average error of the values in the kinetic plots is less than 8%.

Since g-C_3_N_4_ as a photoactive semiconductor is capable of oxidative degradation of various organic pollutants even in the absence of oxidizing additives, photocatalytic experiments were carried out without H_2_O_2_, for comparison, with both pristine and copper-modified catalysts. Interestingly, as Figure 7a shows, the two decay curves hardly differ from each other, which is confirmed by the pseudo-first-order rate constants obtained from the corresponding logarithmic plots (Figure 7c). Since copper may promote the Fenton reaction, which is a thermal process taking place in the dark, the experiments were carried out in the presence of H_2_O_2_ but without irradiation. In this case, the difference between the degradation effects of the catalysts was clearly perceptible (Figure 7b), confirmed by the values of the corresponding rate constants (Figure 7d). The rate constant with the copper-modified catalyst (3% Cu/g-C_3_N_4_) was more than 4 times higher than that with the pristine g-C_3_N_4_.

These results unambiguously indicate that the Fenton reaction is effective in the presence of the copper-doped catalyst. This observation suggests that the oxidation state of Cu in this system is predominantly +1, which is in accordance with the result of the XPS analysis of the 3% Cu/g-C_3_N_4_ catalyst (see Section 2.4).

**Figure 8:**
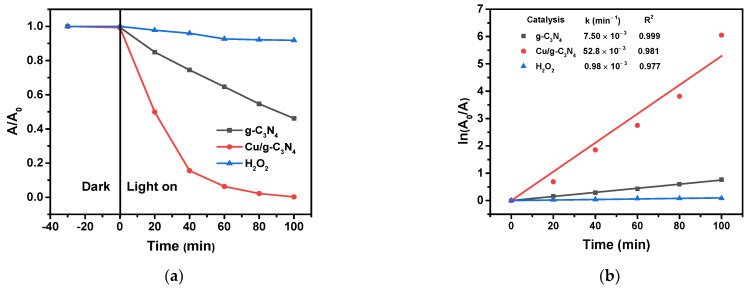
Photoinduced degradation of 4-NP under different experimental conditions: (**a**) light with H_2_O_2_; only H_2_O_2_ (10 mM); pure g-C_3_N_4_ (1.0 g/L, 10 mM H_2_O_2_); or 3% Cu/g-C_3_N_4_ (1.0 g/L, 10 mM H_2_O_2_), (20 ppm 4-NP, pH 3). (**b**) The corresponding logarithmic curves for the determination of rate constants. The average error of the values in the kinetic plots is less than 8%.

Another series of experiments was carried out for the comparison of the degradation effects of H_2_O_2_ alone and together with the photocatalysts under irradiation (Figure 8).

H_2_O_2_ alone caused only a very small decrease in the concentration of 4-NP, indicating that its oxidation reaction with the model pollutant at these concentrations is very slow. (Notably, the applied light source cannot excite H_2_O_2_). In the presence of the unmodified catalyst, the decay rate constant was about 7.5 times higher than without g-C_3_N_4_ (Figure 8b), and more than 3 times higher than the rate determined in the corresponding irradiated system without H_2_O_2_. These significant differences suggest that the presence of H_2_O_2_ synergistically increases the photocatalytic degradation effect of the pristine g-C_3_N_4_ catalyst. However, a far more striking effect of H_2_O_2_ was observed in the case of the photocatalysis with the copper-modified catalyst (Figure 8). The presence of 3% Cu/g-C_3_N_4_ increased the degradation rate constant over 50 times related to that obtained in its absence. This rate constant (5.2 × 10^−2^ s^−1^) is about 7 times higher than in the case of g-C_3_N_4,_ and ca. 12 times higher than the effect of the Fenton process in the dark. These data unambiguously indicate that the degradation efficiency of the copper-modified catalysts in the presence of H_2_O_2_ dramatically exceeds any of those obtained at other combinations in these systems. This outstanding degradation efficiency can be attributed to effects that proved to be synergistic in this combination. The copper species on the surface of the g-C_3_N_4_ function as the conduction-band-electron, trapping and transmitting sites for the appropriate electron acceptor in the solution phase. In the presence of Cu NPs, a Schottky barrier is formed at the interface of the Cu NPs and g-C_3_N_4_, in which photogenerated electrons (e_CB_**^−^**) are transferred to the Cu NPs (Equation (3)) [46].

g-C_3_N_4_ (e_CB_^−^) + Cu NPs → Cu NPs (**^−^**) (3)

Notably, in our case, copper is mostly in a +1 oxidation state, which, however, does not diminish its electron-trapping effect. In the presence of H_2_O_2_, a hydroxyl radical is formed according to the following equation:

e**^−^** + H_2_O_2_ → HO^•^ + HO**^−^** (4)

Hence, this combination ensures a very efficient formation of highly oxidative HO^•^ radicals, because the Cu species as electron-trapping sites diminish the recombination of the photogenerated electron-hole pairs, promoting at the same time the capture of the trapped electrons by the H_2_O_2_ molecules, which produce hydroxyl radicals. This is a synergistic combination, because its efficiency is almost an order of magnitude higher than the sum of the individual efficiencies of these two components, even additionally taking the effect of the Fenton reaction (in the dark), too, into account. The generation of hydroxyl radicals in the presence of H_2_O_2_ was clearly detected by the application of coumarin, utilizing the specific emission of the 7-hydroxy-coumarin derivative formed [47]. According to the experimental results, the formation of hydroxyl radicals could be proved already in the dark, in the presence of 3% Cu/g-C_3_N_4_, but irradiation of the system resulted in a substantial increase in the accumulation of 3% Cu/g-C_3_N_4_, confirming the enhanced production of HO^•^ radicals (Figure S8). Dissolved oxygen can also scavenge the photogenerated electrons to produce O_2_^•−^; the rate constant of this reaction is 1.9 × 10^10^ M^−1^ s^−1^, and the corresponding value for H_2_O_2_ is of about the same order of magnitude (1.1 × 10^10^ M^−1^ s^−1^) [48]. However, the solubility of oxygen in water is only 0.28 mM at 20 °C, while the concentration of H_2_O_2_ in these experiments was of the order of 10 mM. Hence, the superoxide radical anions can play only a minor role in the degradation of 4-NP in our systems containing hydrogen peroxide.

**Figure 9:**
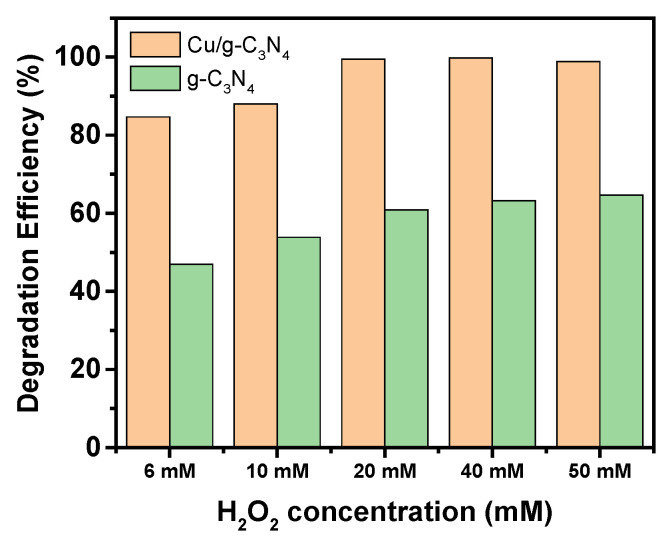
Effect of H_2_O_2_ concentration on the photocatalytic degradation of 4-NP (20 ppm), (1.0 g/L 3% Cu/g-C_3_N_4_, pH 3, 100 min irradiation time).

As for the dependence of the degradation efficiency on the H_2_O_2_ concentration, it shows a monotonously increasing tendency in the range up to 20 mM (Figure 9). However, the efficiency levels above this concentration reach almost 100%, due to the very effective production of hydroxyl radicals.

One may consider the direct oxidation of 4-NP by the holes formed in the valence band. For a significant role of this reaction in the transformation of the model pollutant, both its efficient adsorption on the catalyst surface and a successful competition for the holes with the adsorbed water molecules or HO^−^ ions are needed. According to the results shown in Figure 7a, the adsorption of 4-NP on the surface of both catalysts is about 5–7%, which may promote direct oxidation with h_vb_^+^. However, the dramatic increase in the formation of HO^•^ radicals (measured by coumarin), along with the enhanced degradation of 4-NP, upon copper modification unambiguously indicates that this hydroxyl radical is the predominant oxidizing species in this system, and the role of h_vb_^+^ is just a minor one, even if it cannot be excluded.

#### 2.8.2. Effect of Doped Copper Content

**Figure 10:**
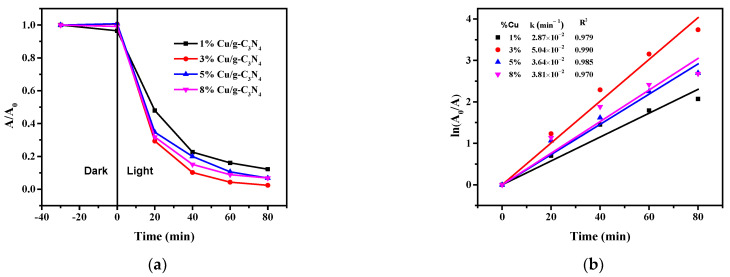
Effect of the Cu content on the photocatalytic degradation of 4-NP (**a**) and the corresponding logarithmic curves for the determination of rate constants (**b**), (20 ppm 4-NP, 20 mM H_2_O_2_, 1.0 g/L, pH 3). The average error of the values in the kinetic plots is less than 8%.

**Figure 11:**
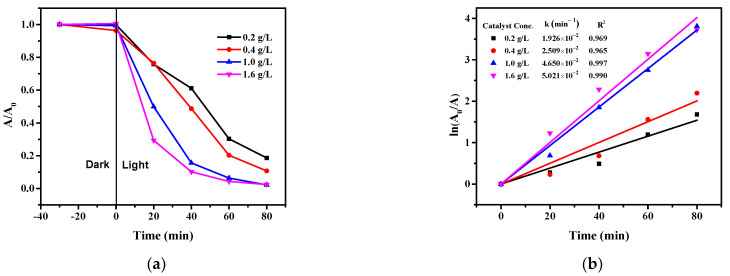
Effect of the 3% Cu/g-C_3_N_4_ concentration on the photocatalytic degradation of 4-NP (**a**), and the corresponding logarithmic curves for the determination of rate constants (**b**), (20 ppm 4-NP, 10 mM H_2_O_2_, pH 3). The average error of the values in the kinetic plots is less than 8%.

Figure 10 indicates that the increase in the copper content of the modified catalyst (Cu/g-C_3_N_4_) enhances the degradation rate only up to 3%. In the 0–3% range, the increase in the pseudo-first-order rate constant is very significant, as shown by the values in Figure 9b and Figure 11b. The corresponding rate constants for 0, 1, and 3% are 7.5 × 10^−3^, 2.87 × 10^−2^, and 5.04 × 10^−2^ min^−1^, respectively. As seen from these data, already 1% (theoretical) copper content increased the degradation efficiency by a factor of about 4 related to the unmodified catalyst, while in the case of 3% doped Cu, this factor became ca. 7. The reason for this dramatic effect in the system containing H_2_O_2_ was interpreted previously in Section 2.8.1.

However, a further increase in the copper content (to 5 and 8%) did not result in higher degradation rates, moreover, it moderately diminished compared to the case of 3% (Figure 10). This effect may be attributed to the relatively high coverage of the catalyst surface by copper particles, thus the light absorption of the g-C_3_N_4_ catalyst becomes less efficient, which cannot be compensated by the electron capturing and transmitting effect of the Cu species.

#### 2.8.3. Effect of the Photocatalyst Concentration

The catalyst concentration can also affect the degradation efficiency under the given experimental conditions. Various 3% Cu/g-C_3_N_4_ contents in the range of 0.2–1.6 g/L were applied with fixed 4-NP and H_2_O_2_ concentrations (20 ppm and 10 mM, respectively) at an optimal pH of 3. The results are displayed in Figure 11.

In the concentration range investigated, a monotonous (but not linear) increase in the degradation rate can be observed. Generally, higher and higher catalyst concentrations resulted in lower and lower proportional enhancements in the rate. This phenomenon may be attributed to the stronger and stronger scattering of light by the colloidal catalyst particles, which competes with the increased absorption of photons inducing photocatalytic degradation. Accordingly, the rate determined at a 1.6 g/L concentration of 3% Cu/g-C_3_N_4_ is just slightly higher than that observed at 1.0 g/L. Hence, for the optimized conditions, also taking economic viewpoints into consideration, a 1.0 g/L catalyst concentration was applied.

This tendency suggests that above a certain catalyst concentration, a further increase will decrease the degradation rate due to the too-high scattering effect. Such a phenomenon was observed in the case of the photocatalytic reduction in Cr(VI) to Cr(III) in the presence of ruthenium-modified g-C_3_N_4_ above a 2.0 g/L catalyst concentration [49].

#### 2.8.4. Effect of the Initial Solution pH

**Figure 12:**
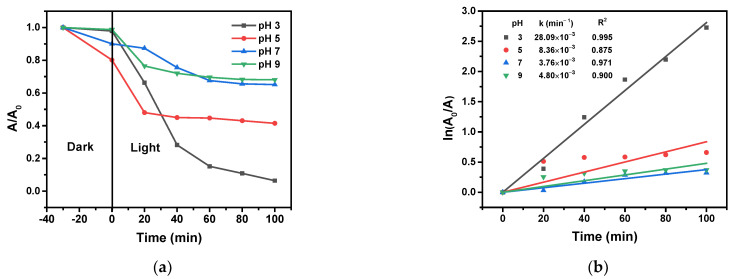
Effect of the initial pH on the photocatalytic degradation of 4-NP (**a**), and the corresponding logarithmic curves for the determination of rate constants (**b**), (20 ppm 4-NP, 10 mM H_2_O_2_, 1.0 g/L 3% Cu/g-C_3_N_4_, pH 3). The average error of the values in the kinetic plots is less than 8%.

Generally, pH considerably affects the semiconductor-based photocatalytic processes, so the effect of pH was studied in the range of 3–9 with 3% Cu/g-C_3_N_4_. In this range, the optimal initial solution pH for the oxidative degradation of 4-NP in the presence of H_2_O_2_ was 3 (Figure 12).

**Figure 13:**
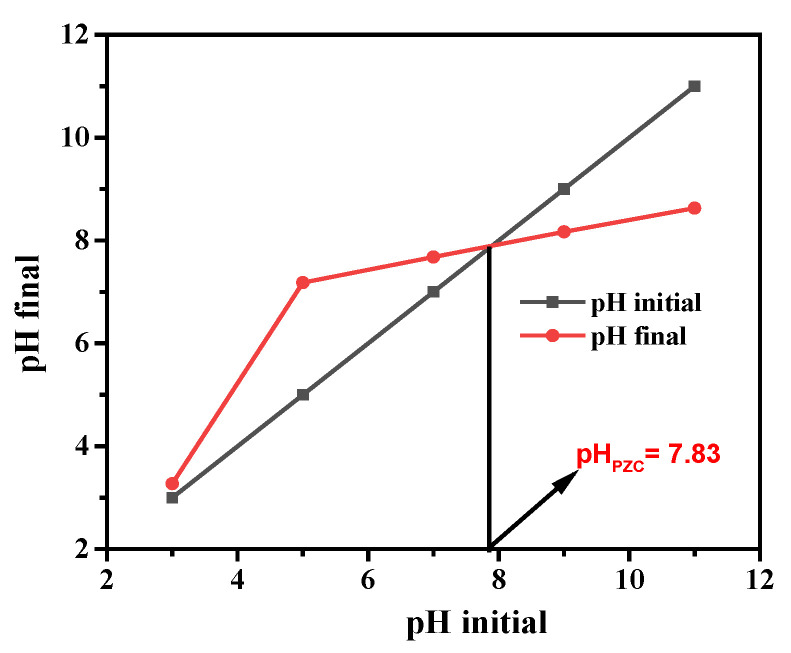
pH_PZC_ of the 3% Cu/g-C_3_N_4_ catalyst.

Since the acidic dissociation of 4-NP is very weak (pK_a_ = 7.15), it hardly exists in the ionic form at pH values below 5. Therefore, in an acidic solution, it occurs as a neutral molecule, the adsorption of which is not affected by the surface charge of the catalyst. The pH value at which the charge of the catalyst surface is zero is designated by pH_PZC_. As Figure 13 indicates, the pH_PZC_ determined for the copper-modified catalyst was 7.83, according to which the catalyst surface at pH = 3 is positively charged. H_2_O_2_ is a very weak acid (pK_a_ = 11.81), thus, it does not dissociate at all at this pH. Hence, its electron scavenging efficiency is much higher than it would be of the corresponding anion (HO_2_^−^). In addition, the reaction between the conduction-band electron and H_2_O_2_ is favored by a higher concentration of protons, which can react with the hydroxide ions formed in this electron scavenging process (see Equation (3)).

#### 2.8.5. Effect of the 4-NP Initial Concentration

**Figure 14:**
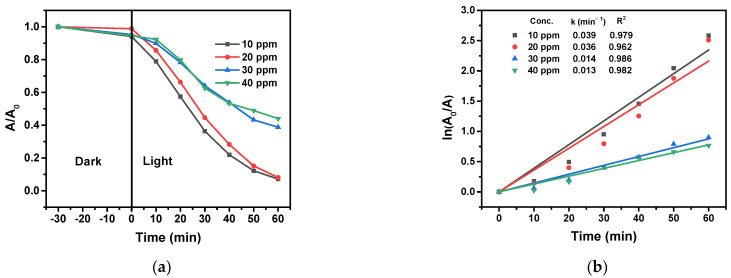
Effect of the initial 4-NP concentration on the photocatalytic degradation of 4-NP (20 ppm) (**a**), and the corresponding logarithmic curves for the determination of rate constants (**b**), (20 mM H_2_O_2_, 1.0 g/L 3% Cu/g-C_3_N_4_, pH 3). The average error of the values in the kinetic plots is less than 8%.

The photocatalytic efficiency of the 3% Cu/g-C_3_N_4_ catalyst in the degradation of 4-NP was investigated at various concentrations (10–40 ppm) of the model pollutant at an initial solution pH of 3.0 and 1.0 g/L of catalyst, with a reaction time of 60 min (Figure 14).

The rate constant for the photocatalytic degradation slightly decreased when the initial concentration of 4-NP was increased from 10 to 20 ppm. Above this concentration, the rate constant significantly dropped. This behavior may have originated from at least two effects, which reduce the efficiency of the production of the oxidizing species (mostly hydroxyl radicals) in the photocatalysis. At higher concentrations of 4-NP, more pollutant molecules can be adsorbed on the surface of the catalyst particles, preventing light absorption during the reaction. In addition, nitrophenol molecules can also scavenge conduction-band electrons (with a rate constant of 4.4 × 10^10^ M^−1^ s^−1^ [48]), in competition with H_2_O_2_. The latter reaction, however, may also contribute to the degradation of 4-NP, even if not as efficiently as the oxidation by hydroxyl radicals. Notably, the absolute rate of the degradation (given by the product of the initial nitrophenol concentration and the rate constant) is the highest at 20 ppm, significantly exceeding the rates at 30 and 40 ppm. In this case, the faster reaction between the hydroxyl radicals and nitrophenol molecules overcompensates the decreased light absorption.

### 2.9. Photodegradation of 4-NP, 2,4-di-NP and 2,4,6-tri-NP

**Figure 15:**
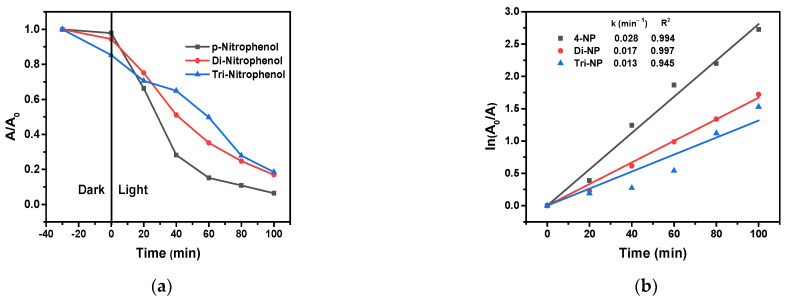
The photodegradation of 4-NP, 2,4-di-NP, and 2,4,6-tri-NP (**a**), and the corresponding logarithmic curves for the determination of rate constants (**b**), (1.0 g/L 3% Cu/g-C_3_N_4_, 20 ppm nitrophenol, 10 mM H_2_O_2_, pH 3). The average error of the values in the kinetic plots is less than 8%.

A series of experiments was carried out to investigate how the number of nitro substituents influences the efficiency of the photocatalytic degradation of nitrophenols under optimized conditions. According to the results obtained (Figure 15), the higher the number of nitro groups on the aromatic ring, the lower the degradation efficiency and the corresponding rate constant.

This observation can be interpreted by the electron-withdrawing effect of the nitro groups. The attack on the aromatic ring by the hydroxyl radical is an electrophilic interaction, and the electron-withdrawing groups decrease the electron density of the carbon atoms available for this attack. Hence, a higher number of nitro substituents results in a lower electron density at the sites of this electrophilic attack, decreasing the efficiency of such an oxidation process.

### 2.10. Reusability of the Photocatalyst

**Figure 16:**
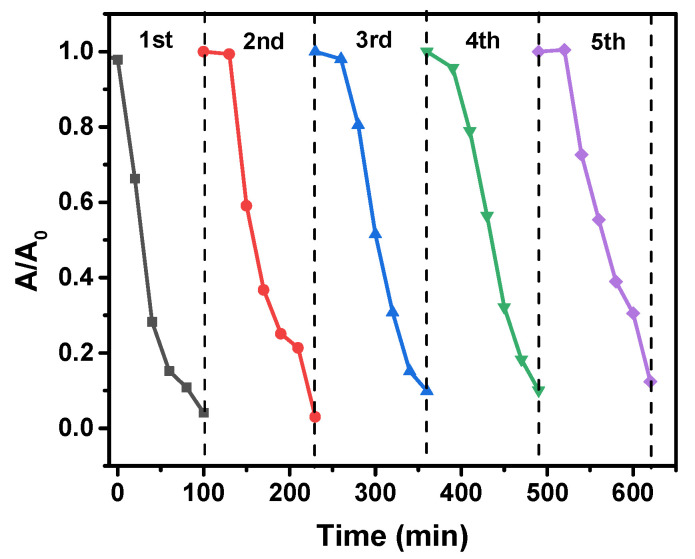
Reusability of the Cu/g-C_3_N_4_ photocatalyst (20 ppm 4-NP, 1.0 g/L 3% Cu/g-C_3_N_4_, 10 mM H_2_O_2_, pH 3). The average error of the values in the kinetic plots is less than 8%.

The reusability of the Cu/g-C_3_N_4_ catalyst was investigated regarding its stability over five consecutive cycles. As Figure 16 displays, the degradation efficiency in the presence of the 3% Cu/g-C_3_N_4_ photocatalyst kept a high value of efficiency in all five experiments; although a slight decrease occurred in the degradation of 20 ppm 4-NP after each run, its value reached 90% even in the 5th cycle. This slight decrease may be attributed to a loss of catalyst during the collection after each irradiation cycle. From the second run, a stagnation period can be observed, during which no or very slow degradation took place. This phenomenon is probably the consequence of the adsorption of the degradation products on the catalyst surface in the previous irradiation, covering the active sites. During the stagnation period, a regeneration of the active sites took place, after which the degradation rate and efficiency became high enough again, ensuring the stability of the catalyst.

## 3. Materials and Methods

### 3.1. Materials

Thiourea (CH_4_N_2_S, 99%) and copper(II) chloride (CuCl_2_·2H_2_O, 99%) were purchased from Merck, Darmstadt, Germany. Sodium borohydride (NaBH_4_, 98%), ethanol (C_2_H_5_OH, 99.7%), sodium hydroxide (NaOH, 99%), and hydrochloric acid (HCl, 36–38%) were obtained from GHTech, Guangzhou, China; 4-nitrophenol (C_6_H_5_NO_3_, 99% from Macklin, Shanghai, China), 2,4-di-nitrophenol (HOC_6_H_3_(NO_2_)_2_, 99% from Aladdin, Mumbai, India), and 2,4,6-tri-nitrophenol (HOC_6_H_2_(NO_2_)_3_, 99.5% from Macklin, China) were used as received.

### 3.2. Catalyst Preparation

#### 3.2.1. Preparation of g-C_3_N_4_ Catalyst

The g-C_3_N_4_ catalyst was prepared by a simple calcination method, which was used in our previous work [49]. First, 3 g of thiourea was heated from 25 to 550 °C at 2 °C min^−1^ for 4 h in an appropriate porcelain crucible with a cover. After the heating procedure, the crucible was cooled down to 25 °C, and the solid sample of the g-C_3_N_4_ catalyst was thoroughly ground to powder and collected as detailed in ref. [50]. The samples obtained in this way were stored in vials. Although g-C_3_N_4_ can be synthesized by hydrothermal condensation from melamine or other triazine precursors, too, in the case of the g-C_3_N_4_ catalyst synthesized from thiourea, which is a sulfur-containing precursor, a sulfur-mediated process improves the degree of polycondensation and polymerization of the g-C_3_N_4_, thus increasing the energy conversion efficiency [51].

#### 3.2.2. Preparation of Cu/g-C_3_N_4_ Catalysts

The Cu/g-C_3_N_4_ catalyst was prepared by a simple method of ultrasonic impregnation at room temperature, using Soner 210H equipment (Rocker Scientific, Taipei, Taiwan) at a frequency of 35 kHz. Initially, 0.57 g g-C_3_N_4_ was homogeneously dispersed in 20 mL of H_2_O in a beaker; then, 6 mL of CuCl_2_ aqueous solution with a concentration of 7.6 mg/mL was slowly added to the beaker. Next, following a 60 min ultrasound treatment, 5 mL of NaBH_4_ solution (60 mg/mL) was slowly added to the mixture, and the solution was kept in ultrasound for another 120 min. Subsequently, the solution was filtered and washed several times with distilled water and alcohol in order to remove impurities. The material obtained was dried at 110 °C for 16 h. Finally, a greenish-brown material was obtained, which was designated as 3% Cu/g-C_3_N_4_.

### 3.3. Sample Characterization

The morphology, microstructure, and optical properties of the photocatalysts prepared in this work were characterized by scanning electron microscopy (SEM, NanoSEM 450 FEI, Eindhoven, The Netherlands) equipped with a TEAM Apollo XL energy dispersive spectroscope (Britain EDAX Co., Ltd., Cambridge, UK), also applying FEI/ThermoFisher Apreo S SEM equipment (Thermo Fisher, Waltham, MA, USA), X-ray diffraction (XRD, Malvern PANalytical, Aeris, Almelo, The Netherlands), Fourier transform infrared spectroscopy (FT-IR, Shimadzu, IRAffinity-1S, Kyoto, Japan), diffuse reflectance UV-Visible spectroscopy (DR/UV-Vis, Carry 5000 UV-Vis-NIR, Santa Clara, CA, USA), and UV-Visible absorption spectroscopy (Agilent 8453, Santa Clara, CA, USA).

X-ray photoelectron spectroscopy (XPS): surface compositions of the samples were determined by a KRATOS XSAM 800 XPS instrument (Kratos Analytical, Manchester, UK). The samples were analyzed by using an unmonochromatized Al K-alpha source (1486.6 eV). All the measurements were conducted in the fixed analyzer transmission (FAT) mode. On each sample, wide-range spectra were collected (at an analyzer pass energy of 80 eV) for surveying the elemental composition. The pass energy of the hemispherical analyzer was set at 40 eV for recording the high-resolution spectra of the C 1s, N 1s, O 1s, Cu 2p, and CuLMM regions. The sp2-bonded C in N=C(-N)_2_, set at 288.2 eV, was used as a reference for charge compensation. The ratio of the elements on the surface was calculated from the integral intensities of the XPS lines using sensitivity factors given by the manufacturer.

For transmission electron microscopy (TEM) analysis, the samples were prepared by depositing a drop of diluted aqueous suspension of the original samples on nickel TEM grids. The grids were covered by continuous carbon amorphous support film. The TEM analyses were carried out by using a Talos F200X G2 instrument (Thermo Fisher Scientific, Eindhoven, The Netherlands), which was operated at 200 kV accelerating voltage, equipped with a field-emission gun and a four-detector Super-X energy-dispersive X-ray spectrometer (Thermo Fisher Scientific). It was capable of working in both conventional TEM and scanning transmission (STEM) modes. STEM high-angle annular dark-field (HAADF) images were collected to visualize the morphology and size of the particles, and STEM-EDS elemental maps were applied to determine and visualize the spatial distribution of elements. We obtained selected area electron diffraction (SAED) patterns in order to identify and characterize the crystalline materials. In the SAED analyses, the camera length was 520 mm.

### 3.4. Photocatalytic Degradation Experiments

The photocatalytic activity of the as-prepared samples of g-C_3_N_4_ and Cu/g-C_3_N_4_ was investigated by measuring the degradation of nitrophenols, mostly 4-NP, under illumination with a 500 W Hg lamp through a cut-off filter transmitting λ ≥ 400 nm. The reaction was carried out at room temperature, in a glass double-shell reactor containing 50 mL of nitrophenol solution (20 ppm) and 1.0 g/L of photocatalyst. The pH of the reaction mixture was adjusted by using 0.1 M HCl or 0.1 M NaOH solutions. The pH values were checked by a pH meter (Hach Sension + PH3, Hach-Lange, Barcelona, Spain). Prior to the light irradiation, the mixture was dispersed by sonication for 3 min and kept for stirring at 250 rpm for 60 min in the dark to attain adsorption–desorption equilibrium. At given intervals, 2 mL of the suspension was taken from the reaction mixture during irradiation and filtered with a syringe filter (0.45 μm nylon membrane) to remove the photocatalyst. The concentration change in the nitrophenol was followed by UV-Vis spectrophotometry, using Agilent 8453 equipment (Santa Clara, CA, USA). The absorbance at 320 nm was applied for the quantitative measurements.

## 4. Conclusions

Copper modification of graphitic carbon nitride (g-C_3_N_4_) and its photocatalytic application for oxidative degradation of nitrophenols in the presence of H_2_O_2_ proved to be a promising method using visible-light irradiation. An outstanding degradation efficacy was achieved because the copper species on the surface of the g-C_3_N_4_ function as active sites for conduction-band-electron trapping (diminishing the recombination of the photogenerated electron-hole pairs) and transmitting to H_2_O_2,_ an efficient electron acceptor, which produces hydroxyl radicals to oxidize nitrophenols. The Fenton reaction has just a minor contribution to the degradation process. Although higher numbers of nitro substituents in these environmentally harmful pollutants, due to the electron-withdrawing effect of this group, decreased the rate of degradation, nevertheless, an efficiency of over 80% was achieved, even in the case of tri-NP during the applied irradiation period. The copper content of the catalyst and the concentrations of the constituents of this photocatalytic system have been optimized. The reusability of the photocatalyst was proven through several cycles. On the basis of our results, this photocatalytic system can be efficiently applied to degrade harmful aromatic water pollutants such as nitrophenols. 

## Figures and Tables

**Table 1:** The calculated conduction band (ECB) and valence band (EVB) energies.

Catalyst	E_g_ (eV)	E_VB_ (eV)	E_CB_ (eV)
g-C_3_N_4_	2.63	1.49	−1.14
Cu/g-C_3_N_4_	2.56	1.45	−1.11

## Data Availability

The data presented in this study are available on request from the corresponding author. The data are not publicly available due to privacy.

## Supplementary Materials

The following supporting information can be downloaded at: https://www.mdpi.com/article/10.3390/molecules28237810/s1, Figure S1: SEM images of catalyst materials 3% Cu/g-C_3_N_4_ (a) and g-C_3_N_4_ (b) at 10,000× magnification; Figure S2: EDS spectrum of the 3% Cu/g-C_3_N_4_ sample (SEM); Table S1: XPS surface composition (at.%) of the 3% Cu/g-C_3_N_4_ sample; Figure S3: The C 1s binding energy region in the XPS of the 3% Cu/g-C_3_N_4_ sample; Figure S4: The N 1s binding energy region in the XPS of the 3% Cu/g-C_3_N_4_ sample; Figure S5: The Cu 2p binding energy region in the XPS of the 3% Cu/g-C_3_N_4_ sample; Figure S6: Cu LMM Auger peak and modified Auger parameter of the 3% Cu/g-C_3_N_4_ sample; Figure S7: EDS spectrum of the 3% Cu/g-C_3_N_4_ sample (TEM); Figure S8: Formation of 7-hydroxy-coumarin upon visible-light irradiation in the presence of 3% Cu/g-C_3_N_4_ catalyst. Before starting the irradiation, the reaction mixture was kept in the dark for 5 h.
