# Supplementary material for: Effect of Copper-Modification of g-C3N4 on the Visible-Light-Driven Photocatalytic Oxidation of Nitrophenols
Source: Molecules. 2023 Nov 27;28(23):7810. doi: 10.3390/molecules28237810 (PMC10708227; doi:10.3390/molecules28237810)
Supplement: Supplementary file 1 [file molecules-28-07810-s001.zip › molecules-2725928-supplementary.pdf]

Supplementary Materials

for

Effect of Copper-Modification of g-C_3N_4 on the Visible-Light-Driven Photocatalytic Oxidation of Nitrophenols

Truong Nguyen Xuan [1], Dien Nguyen Thi [2], Quang Tran Thuong [1], Tue Nguyen Ngoc [1], Khanh Dang Quoc [3], Zsombor Molnár [4], Shoaib Mukhtar [5], Erzsébet Szabó-Bárdos [5], and Ottó Horváth [5,*]

[1] School of Chemistry and Life Sciences, Hanoi University of Science and Technology, No.1 Dai Co Viet street, Hai Ba Trung distric, Hanoi 100000, Vietnam; truong.nguyenxuan@hust.edu.vn (T.N.X.); quang.tranthuong@hust.edu.vn (Q.T.T.); tue.nguyenngoc@hust.edu.vn (T.N.N.)
[2] Viettel Aerospace Institute, Viettel Group, Hoa Lac high-tech park, Thach That distric, Hanoi 10000, Vietnam; diennt15@viettel.com.vn (D.N.T.)
[3] School of Materials Science and Engineering, Hanoi University of Science and Technology, No.1 Dai Co Viet street, Hai Ba Trung distric, Hanoi 100000, Vietnam; khanh.dangquoc@hust.edu.vn
[4] Environmental Mineralogy Research Group, Research Institute of Biomolecular and Chemical Engineering, University of Pannonia, H-8210 Veszprém, POB. 1158, Hungary; molnar.zsombor@mk.uni-pannon.hu (Z.M.)
[5] Research Group of Environmental and Inorganic Photochemistry, Center for Natural Sciences, Faculty of Engineering, University of Pannonia, P.O.B. 1158, Veszprém H-8210, Hungary
* Correspondence: horvath.otto@mk.uni-pannon.hu; Tel+36-88-624-000 / 6049 ext

---

Table of content

Figures S1 and S2, Table S1 2
Figures S3 and S4 3
Figures S5 and S6 4
Figures S7 and S8 5

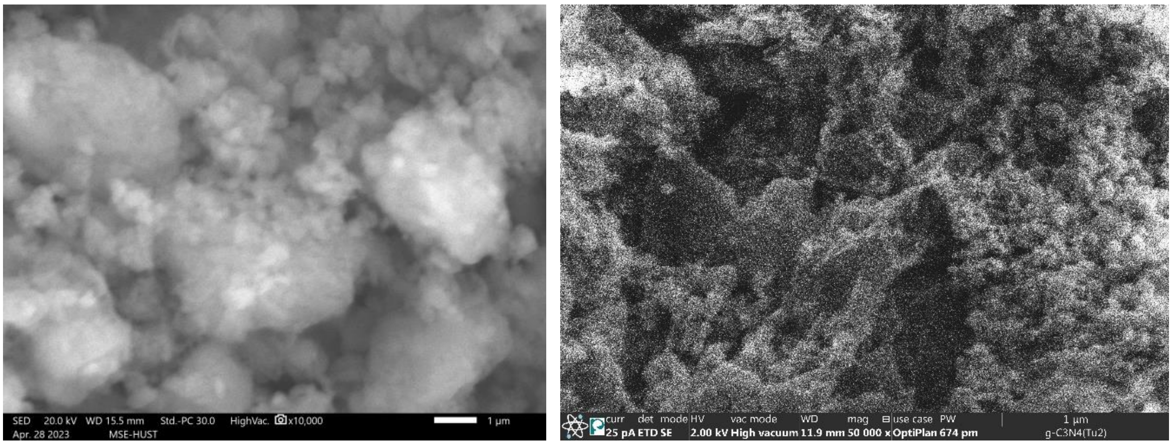

(**a**) (**b**)

Figure S1. SEM images of catalyst materials 3% Cu/g-C_3N_4 (**a**) and g-C_3N_4 (**b**) at 10 000× magnification.

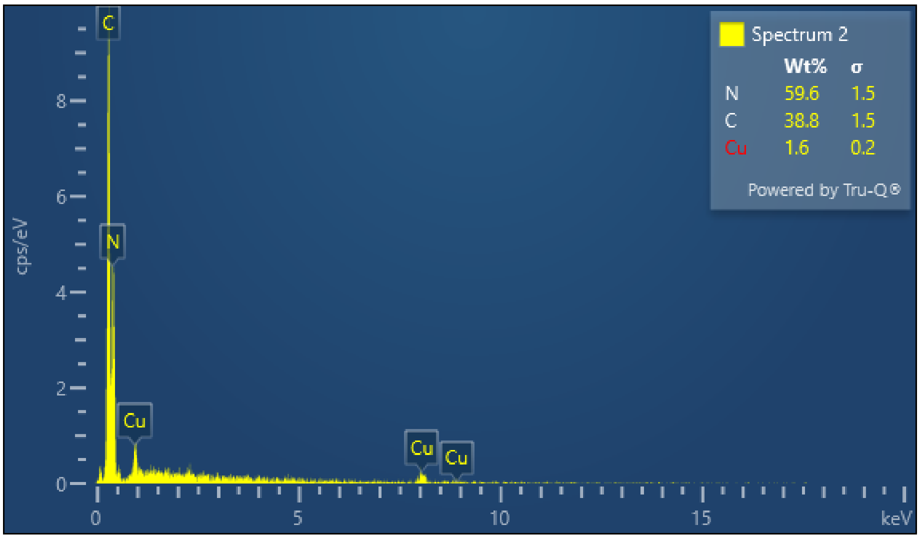

Figure S2. EDS spectrum of the 3% Cu/g-C_3N_4 sample (SEM).

Table S1. XPS surface composition (at.%) of the 3% Cu/g-C_3N_4 sample.

Element	Surface ratios (at.%)
O 1s	39.9
N 1s	51.8
C 1s	39.9
Cu 2p	3.7
N/Cu	14.0

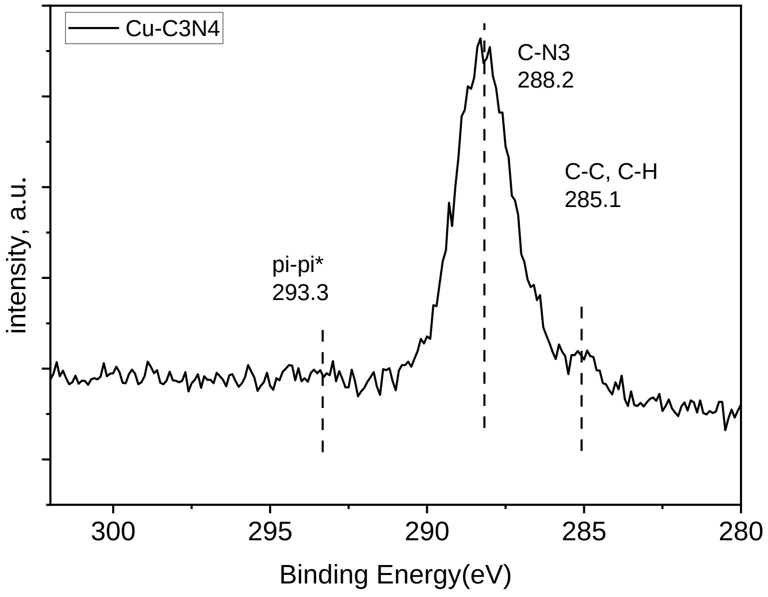

Figure S3. The C 1s binding energy region in the XPS of the 3% Cu/g-C_3N_4 sample.

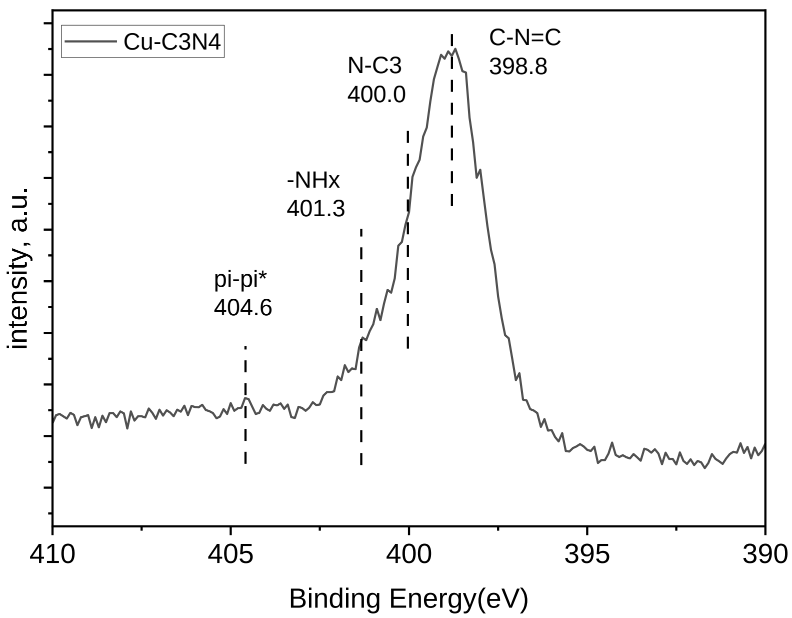

Figure S4. The N 1s binding energy region in the XPS of the 3% Cu/g-C_3N_4 sample.

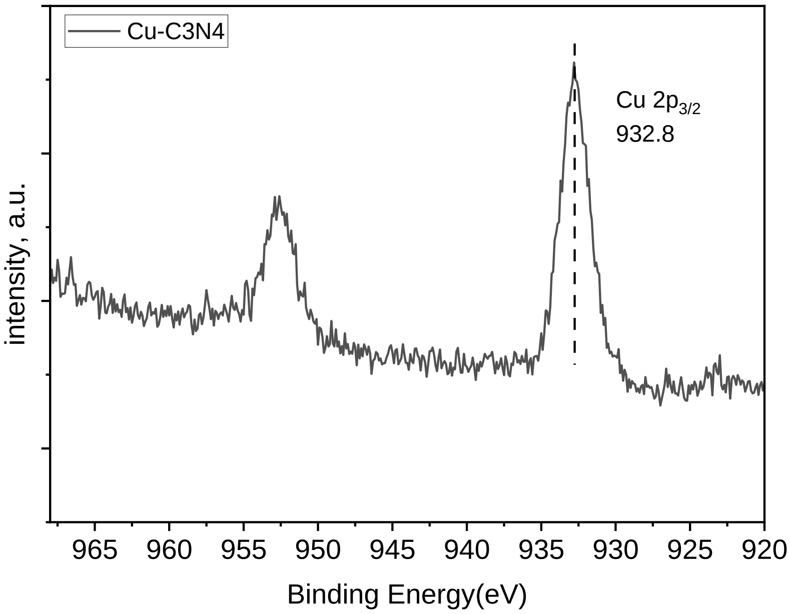

Figure S5. The Cu 2p binding energy region in the XPS of the 3% Cu/g-C_3N_4 sample.

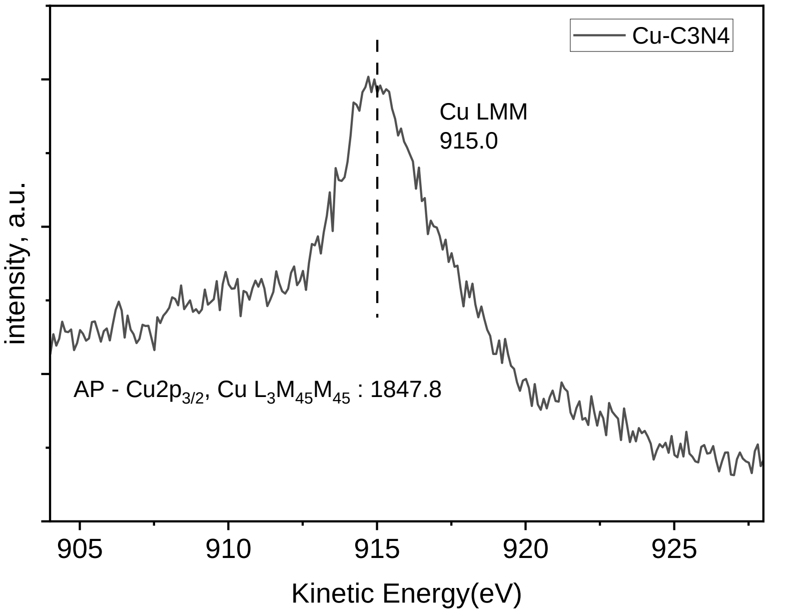

Figure S6. Cu LMM Auger peak and modified Auger parameter of the 3% Cu/g-C_3N_4 sample.

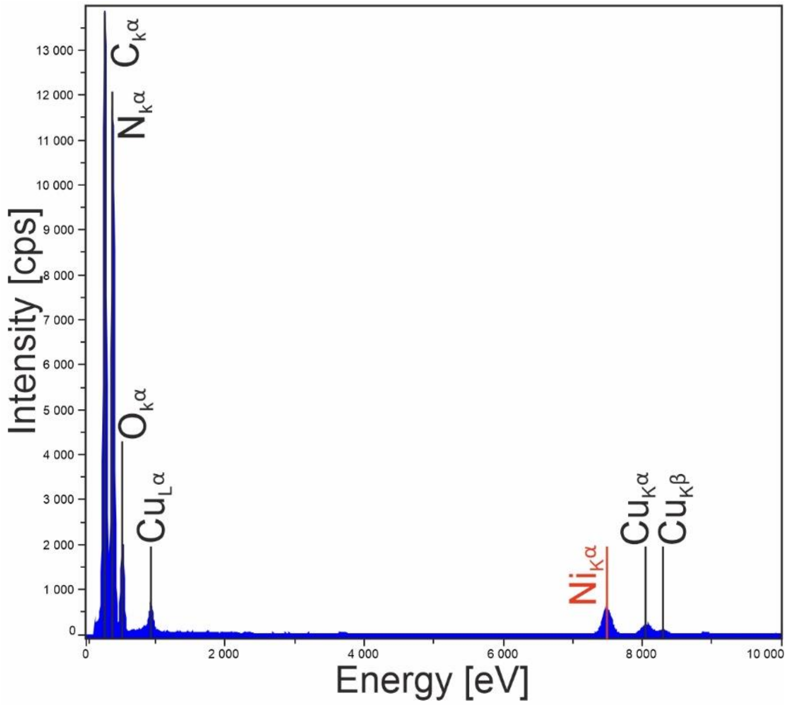

Figure S7. EDS spectrum of the 3% Cu/g-C_3N_4 sample (TEM).

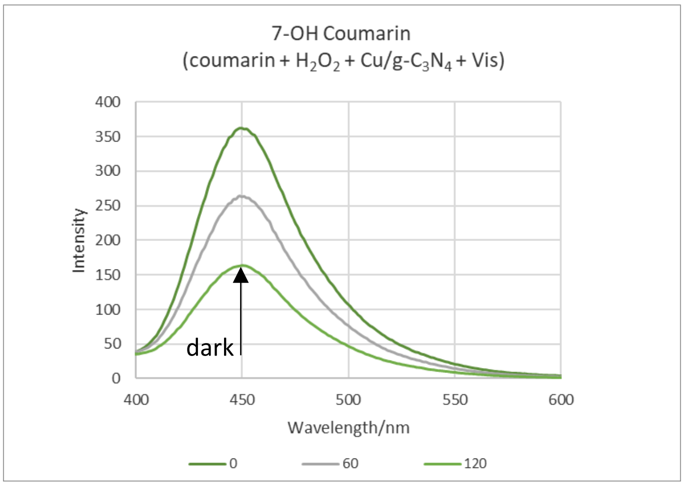

Figure S8. Formation of 7-hydroxy-coumarin upon visible-light irradiation (for 120 min) in the presence of 3% Cu/g-C_3N_4 catalyst. Before starting the irradiation, the reaction mixture was kept in dark for 5 hours.
